# Feature Selection and Model Optimization for Survival Prediction in Patients with Angina Pectoris

**DOI:** 10.3390/jcm14228111

**Published:** 2025-11-16

**Authors:** Róbert Bata, Amr Sayed Ghanem, Attila Csaba Nagy

**Affiliations:** Department of Epidemiology, Faculty of Health Sciences, University of Debrecen, H-4032 Debrecen, Hungary; bata.robert@etk.unideb.hu (R.B.); aghanem@etk.unideb.hu (A.S.G.)

**Keywords:** survival analysis, machine learning, feature selection, EHR, comparative study, angina pectoris, type 2 diabetes mellitus

## Abstract

**Background:** With the rapid emergence of novel survival models and feature selection methods, comparing them with traditional approaches is essential to define contexts of optimal performance. **Methods:** This study systematically evaluates nine survival models combined with nine feature selection methods for predicting the occurrence of angina pectoris using electronic health record (EHR) data from a Hungarian hospital (*n* = 29,655, features = 1150). Performance was assessed with the concordance index (C-index) and integrated Brier score (IBS) to compare predictive accuracy across methods. **Results:** Tree-based survival models, particularly gradient-boosted survival (GBS) and random survival forest (RSF), consistently outperformed conventional approaches in terms of C-index, but showed slightly worse calibration as reflected in their higher IBSs. The best-performing model was RSF, which was optimized using Bayesian hyperparameter tuning. For feature selection, tree-based methods such as Boruta and RSF-based approaches showed superior performance. We further identified clusters of feature selection methods and generated consensus feature sets. We also analyzed the internal relationships between the selected features. Survival model performance was also examined over time using the time-dependent Area Under the Curve (AUC) based on the best-performing feature set. **Conclusions:** Our findings highlight the substantial impact of recent methodological innovations in survival analysis, which offer significant gains in predictive accuracy and efficiency, ultimately support more robust clinical decision-making in the early identification of angina pectoris among patients with diabetes.

## 1. Introduction

Accumulating raw data in electronic health records (EHRs) creates both a need and an opportunity to employ various predictive methods. These methods aim to improve health outcomes through early disease detection, timely intervention, and personalized patient management [1,2]. Survival analysis methods are among the most widely used techniques for estimating the probability of an event, such as death or disease occurrence, happening over a period of time. The unique aspect of survival methods is their ability to handle censored data when the event of interest does not occur during the examined timespan but might come up later on. The Cox proportional hazards (COX) model has been used for a long time for survival prediction complemented with Kaplan–Meier curves to estimate the observed event occurrences during time [3]. With the emergence of modern machine learning techniques, a new class of methods has been introduced alongside traditional approaches, offering the capability to capture complex, non-linear relationships among features in a more flexible manner. However, these machine learning methods often sacrifice interpretability, functioning as ‘black boxes’, whereas traditional statistical models, such as the COX model or Weibull regression (Weibull), remain popular due to their ability to provide easily interpretable and clinically informative results.

The ‘curse of dimensionality’ presents significant challenges when dealing with high dimensional data, like sparsity of data, computational complexity, model overfitting, difficulty in recognizing meaningful patterns, and reduced interpretability [4]. To address the challenges associated with high dimensionality, various feature reduction methods can be employed, including dimension reduction techniques which combines new features in fewer dimensions from the existing ones (e.g., Principal Component Analysis (PCA) and t-distributed Stochastic Neighbor Embedding (t-SNE)), and feature selection methods, which select a subset of the original feature set without transforming the original features (e.g., Lasso and Boruta) [5]. Feature selection methods were applied in this research to address high dimensionality while preserving the interpretability of the contribution of the original features to the survival model.

To date, a few studies have comprehensively compared the performance of various survival analysis models [6,7,8,9], and even fewer have evaluated survival models in combination with different feature selection methods [10,11,12,13,14,15]. Among the identified studies, only two provided comprehensive evaluations across a wide variety of models and feature selection techniques [10,11]. Although survival analysis has been extensively researched, significant differences in findings persist across various studies. For instance, Spooner et al. identified the Cox-Boost method as the best performing survival model [10], whereas Kolasseri et al. found random survival forest (RSF) to have superior performance compared to other models [6]. Differences are even more pronounced regarding feature selection methods. Leger et al. highlighted mutual information-based methods (mRMR, MIM) as the best-performing approaches [11], while Spooner et al. ranked minimum redundancy maximum relevance (mRMR) as the least effective method [10]. Our research addresses these inconsistencies by introducing a novel perspective in comparing survival models and feature selection techniques. Specifically, we incorporated previously unexplored feature selection methods, such as Bayesian Ridge Regression (Bayesian Ridge) and Boruta, alongside advanced survival models like RSF optimized through Bayesian hyperparameter tuning. Additionally, we analyzed the internal relationships among the selected features, identified clusters of feature sets based on homogeneity, and evaluated the time-dependent performance of survival models applied to the optimal feature selection method. Our findings clarify comparative performance and practical trade-offs among different survival models and feature selection approaches, contributing a more comprehensive understanding to the existing literature.

The purpose of this study was to compare the performance of different survival models across various variable sets assembled using multiple feature selection methods, and to evaluate the feature selection methods themselves, including a comparison of the resulting feature sets with respect to each other. We performed a longitudinal analysis to predict the occurrence of angina pectoris, coded according to the International Classification of Diseases, 10th Revision (ICD-10: I20), after an initial diagnosis of type 2 diabetes mellitus (ICD-10: E11). In shaping the comparative analysis framework, our underlying aim was to establish an objective and fair environment for comparing survival models and feature selection methods. The results of the research are discussed in three sections. In the first, nine feature selection methods are compared based on the sets of selected features. In the second, the performance and stability of nine survival models are evaluated and compared using the concordance index (C-index) and integrated Brier scores (IBS). Finally, in the third, the performance of the survival models is assessed over time using the optimal feature set identified in the previous phases, allowing the evaluation of each model’s predictive ability in time.

## 2. Materials and Methods

### 2.1. Dataset and Data Preprocess

The data were collected from the health records of the Clinical Center of the University of Debrecen between 2007 and 2021. The dataset contains information derived from patients’ yearly medical visits; therefore, a single patient may contribute multiple years of data. For this analysis, we aggregated the data on a yearly basis, summarizing laboratory measurements using the median and recording the occurrence of comorbidities as binary indicators. This approach ensured that each patient was represented by a consistent yearly record in the survival models. Patients diagnosed with type 2 diabetes mellitus (E11) were excluded from the study if angina pectoris (I20) occurred prior to the diagnosis of diabetes. In the final dataset 14,921 patients with E11 were included (*n* = 29,655 observations). The event of interest (I20) occurred in 3225 cases (10.87%). The maximum follow-up period was 15 years. The original feature set contains 1150 variables.

To prepare a tractable feature set from the high-dimensional and sparsely populated data, a multi-level preprocessing phase was employed. Features with low variance (variance < 0.01) were excluded to eliminate near constant variables with little discriminatory power. Next, the problem of multicollinearity was addressed by calculating the Variance Inflation Factor (VIF) on the remaining features, to iteratively remove the variables with high multicollinearity (VIF > 5). Finally, all retained continuous features were standardized using z-score normalization. The last two steps were introduced to stabilize model fitting procedures since significant multicollinearity and large differences in feature scaling can lead to convergence issues in parametric (Weibull) and semi-parametric (Cox) approaches that were utilized during comparative process. The study dataset consists of 198 features, including ICD codes recorded in patients’ health records, laboratory test results, and demographic variables such as age and gender. In addition, the dataset contains the essential variables for survival analysis: the follow-up time, defined as the period from the diagnosis of type 2 diabetes mellitus (E11) to the occurrence of angina pectoris (I20) or censoring, and the event indicator, which denotes whether I20 was diagnosed during follow-up.

### 2.2. Study Design

A common threshold was established for evaluating the feature selection methods, based on the number of features retained by Lasso [16] and Boruta [17]. These two methods are not directly parametrizable with respect to the number of selected features, as they inherently determine feature set size based on their internal selection criteria. Since they employ fundamentally different strategies, with Lasso leveraging linear associations between predictors and outcomes and Boruta capturing non-linear and interaction effects, the threshold was defined to span both statistical and heuristic perspectives. When applied to the full dataset of 1150 variables, Lasso and Boruta initially retained 69 and 60 features, respectively. After applying a preliminary variance filter (198 variables), these numbers became 61 for Lasso and 51 for Boruta. To enable a fair and unbiased comparison across all feature selection methods, we initially selected 60 as the number of retained features, which lies within the range consistently identified by both techniques. Leger et al. [11] have emphasized that the Cox proportional hazards model remains a strong baseline for time-to-event analysis due to its simplicity and performance comparable to more complex methods. Following this rationale, we first established a baseline Cox model using features selected by Boruta and Lasso, and then conducted a sensitivity analysis to validate the initially selected 60-feature threshold and confirm its suitability as the optimal feature set size. Feature subsets of 40, 60, and 80 variables were evaluated, and the 60-feature configuration emerged as the optimal choice, providing the best balance between model complexity and predictive accuracy. It consistently resulted in a higher C-index and lower IBS than the smaller or larger feature sets, suggesting reduced overfitting and greater model stability (see Supplementary Table S1) Consequently, the number of selected features was set to 60, prioritizing the highest-ranked variables based on log-transformed *p*-values, absolute coefficient values, non-zero coefficients, mutual information (MI) scores, mRMR scores, and permutation importance depending on the feature selection method used [18,19]. To improve the robustness of the findings, a 5-fold stratified cross-validation approach was employed. Although each patient contributes multiple yearly records to the dataset, the outcome event (angina pectoris, I20) is defined once per individual. Accordingly, yearly records were aggregated and used as historical predictors. To prevent data leakage, cross-validation was performed at the patient level so that no individual contributed data to both the training and testing sets. Within each fold, different feature selection methods were directly embedded into the training process of the survival models, ensuring that each model was trained and evaluated on distinct data subsets. This approach maintained consistency by selecting largely stable subsets of features across the various survival models. Consequently, this methodology effectively mitigated overfitting and enabled fair comparisons between the different feature selection techniques integrated into survival analysis models.

The selected survival models’ performance on the diverse feature sets were compared objectively with the computed *C-index* [20] and *IBS* [21]. *C-Index* works by comparing pairs of individuals in the dataset on the basis of the predicted risk scores and the observed survival times. The harmony introduced by the term concordance happens when the event occurs earlier for an individual who has been exposed to a higher risk compared to another individual who has not been exposed to the same level of risk [22]. The *C-index* measures the probability of concordance, indicating how likely it is that two randomly chosen individuals will have correctly ranked risk predictions [22,23]. If the event of interest is not observed within the study period, the time to event relation is right censored, just like in the current study [24]. When comparing two subjects, the fundamental assumption is that their failure times differ, and only the individual with the earlier event time is observed, while the other may be censored [23,25]. Higher *C-index* (closer to 1) indicates better model performance, while a value of 0.5 suggests a model with no discriminative ability (random guessing).

*C-index* is calculated as follows:

$$C-index=\frac{number\;of\;concordant\;pairs+0.5\times (number\;of\;tied\;pairs)}{number\;of\;camparable\;pairs}$$

Brier score (*BS*) is another essential metric in survival analysis. It not only measures discrimination like the *C-index*, but also evaluates calibration and accuracy over time. It quantifies the difference between predicted survival probabilities and actual outcomes, averaged over the follow-up period [26,27]. Better model performance is indicated by lower *BS* values (closer to 0), whereas a score of 0.25 suggests no discriminative ability, meaning the model performs at the level of random guessing. *IBS* is the average of the *BS* values for a specific timeframe, basically a time-weighted *BS* [28].

Brier score is calculated as follows:

$$BS=\frac{1}{n}\sum_{i=1}^{n}\omega_{i}{\left(p_{i}-\delta_{i}\right)}^{2}$$

*n* = number of individuals;

*p_i_* = predicted survival probability for individual I;

δ*_i_* = event indicator (1 if event occurred, 0 if censored).

Integrated Brier score (*IBS*) is calculated as follows:

The *IBS* aggregates the Brier score over a range of time points.

$$IBS=\frac{1}{T}\int_{t_{0}}^{T}BS\left(t\right)\;dt$$

*T* = Maximum follow-up time;

*t*_0_ = Minimum time point considered.

In the equation, *BS(t)* is the Brier score at time *t*, defined as the mean squared difference between the predicted survival probability and the observed event status at that time.

### 2.3. Feature Selection Methods

In the pipeline of data processing, feature selection has an important role not just in survival analysis but in the broader context of data science. It contributes to identify the most relevant variables, which significantly influence the survival outcome and to reduce the dimensionality of the dataset. High-dimensional datasets can introduce noise and redundancy that makes the model and the identifying of the predictors challenging. In the current study, different types of feature selection methods were employed, and their performance was subsequently evaluated using comparative analysis. These methods fall into four main categories: filter methods, wrapper methods, embedded methods, and hybrid methods.

Filter methods evaluate each predictor’s contribution independently with respect to the outcome, meaning that the interactions among the predictors are not accounted for [29]. Univariate Cox fits a Cox model separately for each predictor in order to assess the association with survival. It cannot capture the combined effect of multiple variables. Features are ranked based on the negative log-transformed *p*-values (−log_10_(*p*)), with lower *p*-values indicating stronger associations with survival time [30]. Univariate Mutual Information (Univariate MI) is an information-theoretic metric that measures the dependence of two variables. It tells how much knowing one variable reduces the uncertainty about another. Univariate MI can be used to assess how well a predictor explains variability, and unlike linear correlation based approaches, it can capture non-linear relationships [31]. The features with the highest MI scores were kept after the MI scores were calculated between each predictor and the discretized survival time.

Wrapper methods evaluate subsets of features by training a model iteratively and assessing its performance while adding or removing features based on model performance to identify the most relevant subset [32]. Starting with the entire dataset, recursive feature elimination (RFE) iteratively eliminates the least significant features according to model performance and repeats the process until the desired number of features remains [33]. RFE captures interdependencies among features. Features were ranked based on the absolute values of their coefficients (|β|), as larger coefficients indicate stronger influence on survival.

Embedded methods integrate feature selection directly into the model training process, in contrast to filter and wrapper methods, which operate independently from the learning algorithm [34]. In embedded approaches, important features are identified automatically during model optimization. Lasso regression (Lasso) is a penalized regression method that applies L1 regularization, forcing some feature coefficients to shrink to zero, in this way removing them from the model and eliminating their contribution in the model fitting [16]. Features were selected based on non-zero Lasso coefficients, with event occurrence used as a sample weight to account for censored data. Bayesian Ridge regression (Bayesian Ridge) is a probabilistic approach that automatically learns the optimal regularization strength from the data, unlike standard Ridge regression, which manually sets the regularization parameter (λ) [35]. The survival time was log-transformed to improve regression stability. Feature importance was determined based on the absolute values of the learned coefficients (|β|).

Hybrid methods combine elements of the above-mentioned methods aiming to create balance between the computational efficiency with model-informed feature selection. The minimum redundancy maximum relevance (mRMR) method reduces feature redundancy while optimizing their relevance to the target variable. The ranking of features is based on mutual information, which guarantees that the selected features are non-redundant and informative; however, it does not take predictors interactions into account [36]. Features were selected iteratively with the highest mRMR score. Boruta is a feature selection approach that iteratively compares feature importance against randomly permuted shadow features, retaining only those which consistently achieve higher importance [17]. Boruta effectively manages feature interactions and nonlinear relationships, although it requires considerable computational resources. Gradient-boosted survival (GBS) builds shallow trees incrementally, and optimizes each step to correct the error of the previous trees, while random survival forest (RSF) is an ensemble of fully grown decision trees [37,38]. GBS is a tree-based machine learning approach that sequentially fits a series of weak learners, typically decision trees, to optimize a loss function adapted for censored data, such as the negative log partial likelihood [39]. Its strength lies in capturing complex, non-linear relationships, making it highly suitable for high-dimensional datasets where traditional models may struggle. RSF, in contrast, constructs multiple decision trees using bootstrap samples of the data, employing a log-rank splitting rule to effectively handle censored survival outcomes [40]. Like GBS, RSF is capable of modeling non-linear interactions without strong parametric assumptions. Both methods assess feature importance with permutation importance besides mean decrease in impurity. Permutation importance evaluates how much a feature influences a model’s accuracy by randomly shuffling its values in the test dataset and measuring the change in performance. If the model’s accuracy drops significantly, it means that feature is important. If there is little to no change, the feature likely has minimal impact. This method helps determine which variables contribute the most to predictions by assessing how the model reacts when their order is disrupted. In this manner it is effective for capturing complex interactions and non-linear effects. Features are ranked based on their mean importance scores, which represent the average contribution of each feature to the model’s predictive performance across multiple iterations or trees.

### 2.4. Models for the Survival Analysis

The application of regression-based survival models in time-to-event analysis is essential for estimating hazard functions and modeling the impact of covariates on survival probabilities [3,41]. The *Cox Proportional Hazards* (Cox) model and the *parametric Weibull* (Weibull) model represent two distinct but complementary methodologies for characterizing survival distributions. The former, a semi-parametric approach, circumvents explicit specification of the baseline hazard function, whereas the latter, a fully parametric alternative, imposes a specific hazard functional form that enables extrapolation beyond observed event times [42].

In the Cox model, the hazard function $h\left(t\;|\;X\right)$ for an individual with covariates X is defined as

$$h\left(t|X\right)=h_{0}\left(t\right)\;\exp\left(X\beta \right)$$

where $h_{0}\left(t\right)$ is the unspecified baseline hazard function, and β is a vector of regression coefficients estimated via partial likelihood maximization [43], rendering inference independent of $h_{0}(t)$. The central assumption underpinning the Cox model is proportionality of hazards, which implies that for any two individuals with covariate vectors $X_{1}$ and $X_{2}$, the ratio of their hazard functions remains constant over time:

$$\frac{h\left(t|X_{1}\right)}{h\left(t|X_{2}\right)}=\exp\left(\left(X_{1}-X_{2}\right)\beta \right)$$

This assumption is critical for model validity and is empirically evaluated using Schoenfeld residual diagnostics or time-dependent covariate interactions [44]. While Cox regression provides a robust framework for estimating relative hazard effects, its inability to explicitly model the baseline hazard function precludes direct derivation of survival or cumulative hazard functions, thereby limiting its predictive applicability in certain clinical settings [45].

To address the need for fully specified hazard dynamics, the Weibull model introduces a parametric framework where the hazard function follows the form

$$h\left(t|X\right)=\lambda pt^{p-1}\exp\left(X\beta \right)$$

where λ (scale parameter) and p (shape parameter) govern the temporal evolution of the hazard [46]. The corresponding survival function takes the form

$$S\left(t|X\right)=\exp\left(-\lambda t^{p}\exp\left(X\beta \right)\right)$$

Unlike Cox regression, the Weibull model imposes distributional assumptions that enable parametric extrapolation beyond observed failure times, making it particularly advantageous in risk prediction scenarios [47]. The monotonic nature of the Weibull hazard function, dictated by p, allows it to capture either increasing (p>1) or decreasing (p<1) hazard rates, but precludes flexible hazard trajectories seen in multimodal failure processes.

Parameter estimation in the Weibull is conducted via maximum likelihood estimation, optimizing the log-likelihood function

$$l\left(\beta ,\lambda ,p\right)=\sum_{i=1}^{N}\delta_{i}\log h\left(t_{i}|X_{i}\right)+\log S\left(t_{i}|X_{i}\right)$$

where $\delta_{i}$ is an event indicator [48]. Model selection between Cox and Weibull regression is often adjudicated using Akaike Information Criterion and Bayesian Information Criterion, with the former favoring flexibility and the latter prioritizing parametric efficiency [49].

While the Weibull model excels in structured environments where the hazard follows a known pattern, the Cox model remains preferable when hazard dynamics are complex or unknown. The contrast between these two approaches underlines the trade-off between interpretability, flexibility, and predictive stability, forming the basis for comparison with machine learning-based survival models that can relax proportional hazards assumptions and accommodate high-dimensional interactions [50].

The core idea of survival SVM (SSVM) is to maximize the distance between the support vectors to make a better separation between the classes. For each and every timepoint we construct a hyperplane that is separating the subjects where the event has already occurred from those who are still at risk [51,52]. The hyperplanes share a common orientation determined by a coefficient vector. The role of this vector is the same as the coefficients in a linear model, to determine the influence of the covariates on the separation. The objective is to determine the best coefficient vector that maximizes the separation between the two classes while reducing the penalties of misclassification. Nonlinear relationships can be integrated with the usage of kernel functions [53].

Both RSF and GBS were employed in this study as survival models for risk prediction and feature evaluation. Further methodological details on them can be found in Section 2.3.

In this study, the predictive performance of various survival models was evaluated across different feature sets constructed using multiple feature selection techniques. The models examined included Cox, Weibull, SSVM, GBS, and RSF, with corresponding parameter settings and optimization details provided in the Supplementary Materials.

## 3. Results

### 3.1. Comparison of Feature Sets Across Different Models

A consensus feature set was created for each feature selection method, requiring a feature to appear in at least 60% of cases across folds and survival models. A sensitivity analysis of 40%, 60%, and 80% fold-consensus thresholds confirmed that the 60% level resulted in the most stable and balanced feature sets (Supplementary Tables S2 and S3). By tracking the selected features within each fold, we compiled a final dataset for each method including all five stratified folds for the nine survival models, resulting in 45 feature subsets contributing to the consensus data by method. The resulting consensus feature set sizes were Lasso = 60, RSF = 60, GBS = 59, Bay Ridge = 58, RFE = 55, Boruta = 50, Univariate MI = 28, Univariate Cox = 24, and mRMR = 8.

**Figure 1:**
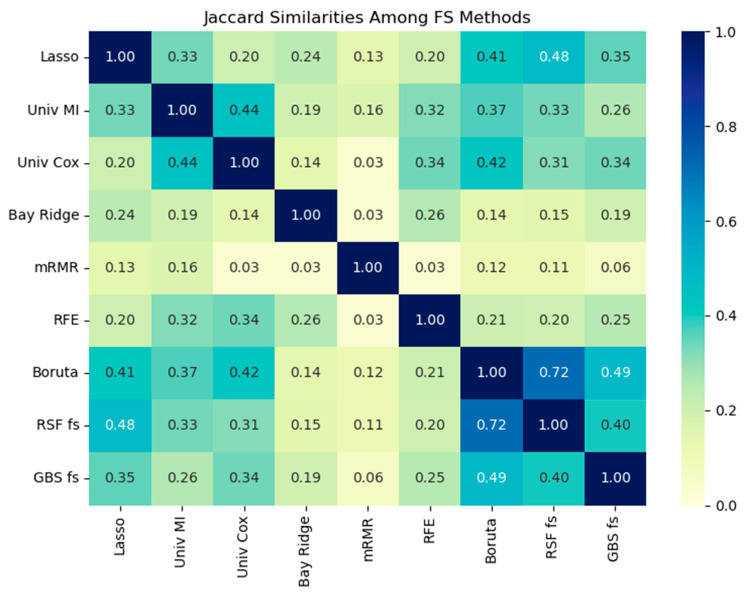
Jaccard Similarity scores among feature selection (FS) methods. In the Jaccard similarity table, the values range from 0 to 1, represented by a gradient from lighter to darker colors. Higher values indicate greater similarity or closer distance. Lasso = Lasso Regression (Least Absolute Shrinkage and Selection Operator); Univ MI = Univariate Mutual Information; Univ Cox = Univariate Cox Regression; Bay Ridge = Bayesian Ridge Regression; mRMR = Minimum Redundancy Maximum Relevance; RFE = Recursive Feature Elimination; Boruta = Boruta Feature Selection; RSF fs = Random Survival Forest Feature Selection; GBS fs = Gradient Boosting Survival Feature Selection.

The Jaccard similarity measure was used to quantify how much overlap exists between the feature subsets selected by different feature selection methods. In other words, it indicates the proportion of shared features relative to the total number of unique features selected across methods. This measure helps assess how consistently the different methods converge on a similar set of relevant variables (Figure 1).

Boruta and RSF has the highest similarity in the entire matrix (0.72). This suggests that Boruta and RSF-based permutation importance select very similar feature subsets. The similarity of RSF and Lasso is relatively high (0.48), indicating a stronger than average overlap. Boruta and GBS also shows moderately high overlap (0.49), suggesting consistency with RSF and GBS. mRMR has particularly low overlaps with most of the methods the largest is a 0.16 with Univariate MI which is still quite low, implying that it selects features distant from the other methods. Bay Ridge is like the Univariate MI has low overlaps with the other methods peaking at 0.26 with the RFE. The similarity measures of Univariate MI, Univariate Cox and RFE range between 0.3 and 0.44 of each other, indicating that they share subsets of features more often than with other methods. Distinct clusters emerge based on the overlap of selected features. Boruta, RSF feature selection, and GBS feature selection show moderate to high similarity, with Jaccard scores above 0.40, whereas methods such as Univariate MI, Univariate Cox, and RFE display only moderate overlap, indicating more variability in the features they select. Lasso interestingly has high overlap with RSF (0.48) and Boruta (0.41). mRMR consistently produces distant features sets compared to the other methods.

**Figure 2:**
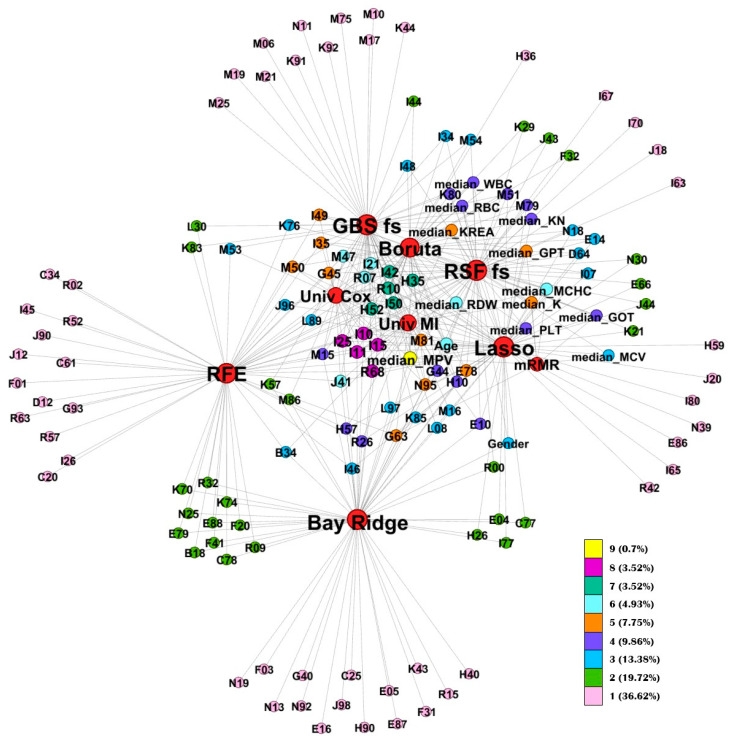
Graph for interrelated features. In the graph, the different colors indicate the occurrence of common elements in the census feature sets. The scale shows how many sets a feature occurs in, and the frequencies are also expressed as percentages. FS methods: Univ Cox = Univariate Cox Regression; Univ MI = Univariate Mutual Information; RFE = Recursive Feature Elimination; Lasso = Lasso Regression; Bay Ridge = Bayesian Ridge Regression; mRMR = Minimum Redundancy Maximum Relevance; Boruta = Boruta Feature Selection; RSF fs = Random Survival Forest Feature Selection; GBS fs = Gradient Boosting Survival Feature Selection.

The internal relationships within the consensus feature sets, based on feature cooccurrences, are represented in the graph (see Figure 2), providing valuable insights into their interdependencies and distribution patterns. There was only one feature (0.70%) appearing in all the nine consensus feature sets, five features appeared in eight sets (3.52%), another five (3.57%) in seven sets, while 36.62% of the features appeared only in one feature set. Roughly 60% percent of the features were connected with some other feature sets.

Within the folds across the survival models the execution time of the feature selection methods was also monitored besides the selected feature sets. The convergence of the feature selection methods from the slowest to the fastest expressed in seconds: GBS (236.17 ± 7.17 s), RSF (225.38 ± 1.05 s), Univariate MI (66.58 ± 1.01 s), Boruta (48.03 ± 0.38 s), mRMR (16.80 ± 0.48 s), Univariate Cox (6.17 ± 0.30 s), RFE (1.29 ± 0.14 s), Lasso (0.76 ± 0.03 s), and Bay Ridge (0.12 ± 0.00 s).

### 3.2. Comparison of Model Performance on the Different Feature Selection Methods

**Figure 3:**
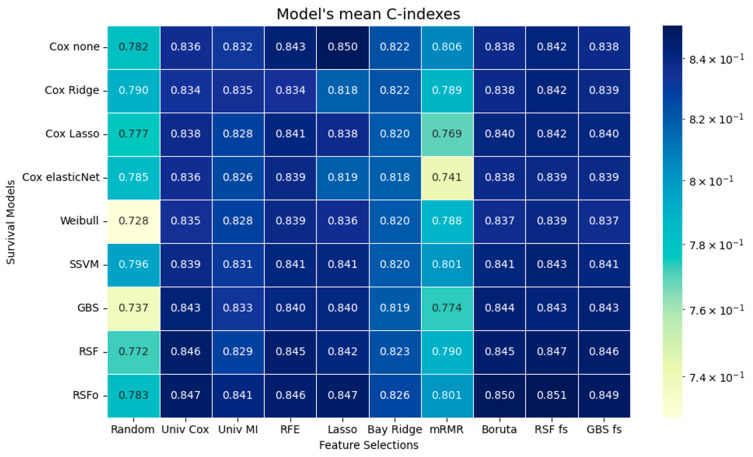
Models’ performance expressed in C-index values. Mean concordance index values are shown for multiple survival models across different feature selection (FS) methods. Cox none = Cox Proportional Hazards without regularization; Cox Ridge = Cox model with Ridge penalty; Cox Lasso = Cox model with Lasso penalty; Cox elasticNet = Cox model with Elastic Net penalty; Weibull = Weibull Regression; SSVM = Survival Support Vector Machine; GBS = Gradient Boosting Survival model; RSF = Random Survival Forest; RSFo = Optimized Random Survival Forest. FS methods: Univ Cox = Univariate Cox Regression; Univ MI = Univariate Mutual Information; RFE = Recursive Feature Elimination; Lasso = Lasso Regression; Bay Ridge = Bayesian Ridge Regression; mRMR = Minimum Redundancy Maximum Relevance; Boruta = Boruta Feature Selection; RSF fs = Random Survival Forest Feature Selection; GBS fs = Gradient Boosting Survival Feature Selection.

The performance of the survival models on each feature set was assessed by averaging the C-index across the cross-validation folds. Besides the discussed feature selection methods random feature selection was included in the table for sanity test but was not evaluated in terms of the other models (Figure 3).

The performance of RSF with Bayesian optimization (RSFo) was very strong across all feature selection methods, especially with the advanced feature selection methods like Boruta, RSF fs, and GBS fs, all within the range of 0.84 to 0.85. The unoptimized RSF was closely following RSFo in performance. The classic Cox variants performed well on the RSF fs and the RFE subsets, where the C-index can approach slightly more than 0.84 with careful feature selection. The parametric Weibull model generally showed lower performance than the more flexible models (like RSF, RSFo, SSVM, and some Cox variants). However, for several feature selection methods, its C-index values were still comparable to the top-performing models and reached values above 0.83. The models’ performance using the features selected with mRMR is only marginally better than that achieved with the randomly assembled features. The standard deviation range of the C-indexes is between 0.004 and 0.01 not including the mRMR feature selection method. mRMR stands out as having consistently higher standard deviation for many models (0.01–0.03). Bayesian Ridge often results in the lowest standard deviation (4 out of 9 models show the smallest standard deviation). The small standard deviation across the models indicating a relatively stable performance. The exception is mRMR, which generally shows higher standard deviation in most models, suggesting that it is less consistent. This derives from the fact that the selected features by the mRMR vary more over the cross-validation folds (see the standard deviations in Supplementary Figure S1).

**Figure 4:**
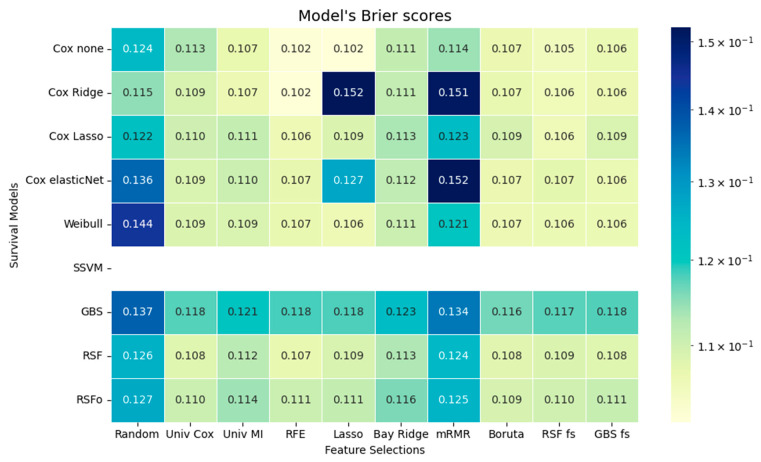
Models’ calibration expressed in integrated Brier scores. Mean integrated Brier scores are presented for multiple survival models across different feature selection (FS) methods. Cox none = Cox Proportional Hazards without regularization; Cox Ridge = Cox model with Ridge penalty; Cox Lasso = Cox model with Lasso penalty; Cox elasticNet = Cox model with Elastic Net penalty; Weibull = Weibull Regression; SSVM = Survival Support Vector Machine; GBS = Gradient Boosting Survival model; RSF = Random Survival Forest; RSFo = Optimized Random Survival Forest. FS methods: Univ Cox = Univariate Cox Regression; Univ MI = Univariate Mutual Information; RFE = Recursive Feature Elimination; Lasso = Lasso Regression; Bay Ridge = Bayesian Ridge Regression; mRMR = Minimum Redundancy Maximum Relevance; Boruta = Boruta Feature Selection; RSF fs = Random Survival Forest Feature Selection; GBS fs = Gradient Boosting Survival Feature Selection.

Cox models showed their strongest performance when combined with RFE and RSF-based feature selections. RSF and RSFo also show reasonable performance, especially with RSF fs and Boruta. In terms of the IBS, the Weibull model improves the performance of the Cox models in certain cases. GBS has consistently higher IBSs than all the other models. The mRMR-selected feature sets result in the highest IBSs overall, suggesting that models trained on these features are more unstable and less well-calibrated, meaning that their predicted survival probabilities deviate more strongly from the observed outcomes. The IBS cannot be calculated for SSVM survival models because they do not produce explicit survival probabilities, only risk scores (Figure 4).

Although the performance differences between the models were generally not substantial, with some exceptions, we used the Friedman Test to identify the best survival model and feature selection method. Among the feature selection methods RSF fs was ranked as the first (*p* < 0.001), and RSFo was ranked as the best performing survival model (*p* < 0.001).

### 3.3. Analysis of Model Performance on the Best Feature Selection Method

**Figure 5:**
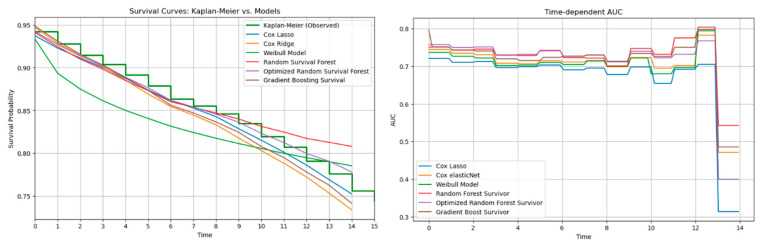
Survival curves and time-dependent Area Under the Curve (AUC).

The predictive performance of the survival models over time was assessed using the optimal feature selection method, RSF fs. First, the survival curves predicted by the models were compared to the observed survival probabilities represented by the Kaplan–Meier survival curve. The Cox variant, GBS, and RSFo models provided good estimations, while the predicted probabilities closely followed the observed probabilities. The Weibull model’s predictions deviated from the observed probabilities by underestimating risk due to its parametric rigidity, which assumes survival declines too quickly. In contrast, the RSF overestimated risk in the last third of the time points. Interestingly, the optimized version of the RSF managed to correct the overly pessimistic survival probabilities at the later timepoints (Figure 5).

Secondly, time dependent Area Under the Curve (AUC) values were plotted for the survival models to evaluate how well a model distinguishes individuals at risk and those not at risk at different timepoints. AUC values remain fairly stable across the timepoints for most models, with slight variation in the AUC values, until the 13th timepoint where they sharply drop. RSF and RSFo have the highest AUC at most timepoints, with GBS closely following them. Cox variants and Weibull tend to have marginally lower AUC compared to other models, suggesting that they are less effective in distinguishing risk groups.

## 4. Discussion

This study introduces several novel aspects that extend beyond traditional survival model comparisons. First, we explored the internal relationships among feature selection methods by examining the overlap of their selected features using Jaccard similarity analysis and graph-based representations. This approach provided insights into how different feature selection strategies relate to one another and helped identify shared or distinct feature patterns across methods.

By monitoring feature selection across cross-validation folds, we constructed consensus feature sets for each method. These consensus sets enabled a systematic comparison of the methods’ stability and their internal feature structures. The Jaccard similarity analysis revealed clear clusters among the methods. For instance, tree-based feature selectors (Boruta, RSF, and GBS) showed high similarity, likely reflecting their shared mechanisms for capturing non-linear and interaction effects among predictors. In contrast, univariate methods (Univariate MI and Univariate Cox) also exhibited mutual similarity, as they independently evaluate each feature’s association with the outcome. Another distinct cluster emerged for methods such as mRMR and Bayesian Ridge, which consistently selected feature sets markedly different from those identified by the other approaches. Importantly, these differences do not imply inferior feature quality but rather reflect divergent selection philosophies; Bayesian Ridge emphasizing shrinkage-based regularization and mRMR prioritizing minimal redundancy. Identifying these clusters provides a practical framework for researchers: rather than evaluating all methods individually, one can select a representative feature selection approach from each cluster to efficiently explore model behavior in new datasets.

The network visualization highlights the internal relationships among consensus features, illustrating how frequently specific variables were retained across different feature selection methods. This analysis revealed a set of core, high-stability predictors that consistently appeared across multiple approaches. The most frequently retained variable was mean platelet volume (MPV), followed by cardiovascular and systemic disease codes such as I25 (chronic ischemic heart disease), I10 (essential hypertension), I42 (cardiomyopathy). Beyond diagnostic codes, laboratory parameters, including red blood cell indices (MCV, MCHC, RDW) and renal function markers (KREA, potassium (K)), along with demographic variables such as age and gender, were frequently selected across methods.

The repeated identification of these features across independent algorithms emphasizes their cross-method robustness and supports their potential as generalizable prognostic indicators for cardiovascular and metabolic risk stratification. These findings align with previous research demonstrating the value of integrating multimodal clinical information for improved cardiovascular risk prediction. In particular, the CAR_2_E_2_ Score study showed that combining clinical, radiographic, and electrocardiographic data enhances the detection of left ventricular hypertrophy and strengthens cardiovascular risk assessment [54]. This parallel supports the clinical relevance of our results, emphasizing that multimodal and data-driven approaches can provide more comprehensive and actionable risk stratification in patient management.

The time-dependent performance analysis compares observed survival with model predictions and their discrimination over time. Tree-based models, especially RSFo and GBS, closely followed the Kaplan–Meier curve and maintained the highest AUC values across follow-up, indicating strong risk discrimination. In contrast, the Weibull model diverged substantially due to its rigid distributional assumption. Despite their superior C-index, tree-based models showed higher IBSs, reflecting less accurate calibration. This trade-off highlights the importance of assessing both discrimination and calibration before clinical application.

Our findings align with those of Spooner et al. and Leger et al., who concluded that there are no major differences in predicative performance among the survival models [10,11]. In agreement with their findings the tree-based survival models generally demonstrated the best predictive performance, despite only minor differences overall. Spooner et al. and Leger et al. specifically identified the Cox-Boost survival method as the best performing model among the tree-based approaches. In our study the GBS aligns with the Cox-Boost method, because it is optimized to the Cox partial likelihood and function in a similar manner to the Cox-Boost method. Not by far but RSFo with Bayesian optimization achieved better predictive performance than the other models; thus, in our case the RSFo outperformed the Cox-Boost method as well.

The tree-based models were followed by the Cox variants, while the parametric Weibull model ranked lowest in predictive accuracy, in accordance with the results of Kolasseri et al. [6]. This may be attributed to its rigidity stemming from the assumption of a predefined probability distribution. However, the tree-based models achieved worse results in terms of the IBS compared to the traditional models like the Cox variants and Weibull. Consequently, if the aim is to rank patients by relative risk, one of the tree-based models should be selected, whereas Cox variants are preferable when the goal is to accurately predict individual survival probabilities. (For recommended use see Supplementary Table S4.) Given that performance differences between traditional and advanced models are minimal, or in some cases, nearly negligible, as shown in the study by Cuthbert et al., where the difference in C-index was only 0.00153, other model characteristics must be considered when selecting an approach [55]. Tree-based methods can detect non-linear relationships, require minimal data preprocessing, although they are more challenging to interpret and have longer execution times. Traditional models, such as Cox regression variants, can only detect linear relationships. However, they are easier to interpret, as the direction and magnitude of associations are directly indicated by the model coefficients. Moreover, traditional methods require careful handling of multicollinearity and data normalization, but offer faster execution times.

Based on the comparative assessment across various feature selection methods, the results support the conclusion of Spooner et al. and Leger et al.: the choice of feature selection method has a greater impact on model performance than the selection of the survival model itself [10,11]. This observation can be interpreted in a broader context, emphasizing that data significantly influences model performance. The survival models performed the worst on the mRMR feature selection method, and the findings are identical with the results of Leger et al., likely because the method selected features with high variability across folds, indicating that the predicative power is highly dependent on the specific subset chosen in each fold. It is clear from the results that using Lasso for feature selection can reduce predictive accuracy when followed by a penalized Cox model. This occurs because Lasso already removes features by shrinking some coefficients to zero, and when a second penalization step is applied in the Cox model, additional useful predictors may be weakened or excluded. As a result, the combined effect can discard variables that would have improved the model’s performance, leading to a loss of important information. Interestingly, survival models, without exception, were performing the best with the tree-based methods (Boruta, GBS, RSF). This finding aligns with Spooner et al., who reported random forest minimal depth among the best-performing approaches, and with Leger et al., who also highlighted tree-based methods as strong performers [10,11].

### 4.1. Clinical Implications and Recommendations

Our findings have several practical implications for clinical research and risk modeling. The results demonstrate that no single survival model consistently outperforms others across all evaluation metrics; rather, the optimal choice depends on the specific clinical objective and the study’s priorities, whether focused on hazard differentiation, interpretability, or discrimination performance. A practical strategy for model selection is to begin with a baseline model, such as the Cox proportional hazards model, and then iteratively evaluate more complex alternatives to identify the best-performing approach for the task at hand.

When the goal is to rank patients by relative risk (e.g., identifying those at elevated risk who may benefit from closer monitoring or preventive therapy), tree-based models such as the RSF and GBS models are advantageous due to their superior C-index values and capacity to capture complex, non-linear interactions. Conversely, when the objective is to estimate individual survival probabilities for counseling, prognosis, or treatment planning, Cox-based models are preferable. Although their discrimination is slightly lower, they offer more accurate and well-calibrated probability estimates, as reflected by lower IBSs.

From a methodological perspective, clustering of feature selection methods provides a practical strategy for simplifying model development. Methods within the same cluster, such as tree-based, shrinkage-based, and univariate approaches, tend to identify similar patterns of predictors. Selecting a representative method from each cluster allows balanced exploration of non-linear, linear, and univariate effects while reducing computational redundancy.

In summary, combining interpretable Cox-based models for accurate probability estimation with flexible tree-based models for enhanced ranking performance provides a balanced and robust framework for patient risk assessment. Simultaneous consideration of C-index and IBS ensures that both discrimination and calibration are optimized, an essential step toward developing survival models that are not only statistically sound but also clinically reliable.

### 4.2. Limitations

This study has several limitations that should be acknowledged. First, the analysis was conducted using data from a single secondary care provider, which may introduce institution-specific bias related to local patient demographics, referral patterns, or diagnostic practices. Consequently, the framework and model performance may not directly generalize to other healthcare settings. To confirm robustness and transferability, external validation on multi-center datasets and prospective testing in independent cohorts are warranted.

Second, ICD coding variability and potential underreporting of comorbidities may have influenced the feature selection process and the models’ ability to capture certain disease patterns. Although standardized coding practices were used, such variability is an inherent limitation of real-world clinical data.

Methodologically, several preprocessing steps, implemented to ensure objective comparison across models, may have constrained model behavior. Variance thresholding applied before modeling could have excluded low-frequency but clinically important predictors, such as rare comorbidities, that contribute little to linear models like Cox regression but could enhance performance in non-linear, tree-based approaches. In addition, low-variance features may have reduced stability and predictive power in some Cox variants, and may have contributed to convergence issues observed in the non-penalized Cox model.

Furthermore, generating cross-validation folds with identical feature subsets improved comparability among survival models but limited the variability of feature selection across folds. This design choice, while necessary for fair model comparison, may have resulted in the omission of potentially relevant features from the consensus set.

Overall, model performance and selection are inherently influenced by the dataset characteristics and feature selection strategy. Future work should aim to validate the framework externally, explore adaptive feature selection across folds, and assess model performance prospectively in broader, real-world clinical populations.

## 5. Conclusions

With the expanding availability of EHRs and advances in analytical techniques, robust survival modeling is increasingly important for translating complex data into actionable clinical insights. In this study, we compared multiple survival models and feature selection strategies using EHR-derived data to evaluate their predictive performance and stability. Our results identified the RSFo as the best-performing model and RSF-based feature selection as the most consistent and generalizable approach. Tree-based models, such as RSF and GBS, excelled in ranking high-risk patients, while Cox-based models provided better-calibrated survival probabilities, making them particularly suitable for individualized risk estimation and clinical decision support. Clinically, these findings support combining interpretable Cox models with flexible tree-based approaches to achieve both accurate prediction and practical usability. Standardizing feature selection and survival modeling pipelines will enhance the reproducibility, interpretability, and clinical relevance of future EHR-based risk prediction models.

## Data Availability

The data that support the findings of this study are available from Clinical Centre of the University of Debrecen, Hungary but restrictions apply to the availability of these data, which were used under license for the current study, and so are not publicly available. Data are, however, available from the authors upon reasonable request and with permission of the Clinical Centre of the University of Debrecen, Hungary.

## Supplementary Materials

The following supporting information can be downloaded at: https://www.mdpi.com/article/10.3390/jcm14228111/s1, Figure S1: The standard deviation for the mean C-indexes; Table S1: Sensitivity analysis of feature set size on model performance. Comparison of mean ± standard deviation for the concordance index (C-index) and integrated Brier score (IBS) across feature set sizes (40, 60, and 80) for the baseline Cox model using Boruta and LASSO-based feature selection. The 60-feature configuration resulted in the most favorable balance between discrimination and calibration performance, supporting its selection as the common threshold for subsequent analyses; Table S2: Sensitivity analysis of fold-consensus thresholds on the number of retained features across all feature selection methods. The table summarizes the mean ± standard deviation of feature numbers retained at different fold-consensus thresholds (40%, 60%, 80%) used to define stable features across cross-validation folds. Increasing the consensus threshold reduced the number of retained features, indicating progressive exclusion of fold-specific variables. The 60% consensus level resulted in an intermediate and balanced subset size, supporting its selection as the baseline threshold for defining reproducible features across folds; Table S3: The table reports the mean ± standard deviation of the number of retained features for each feature selection method across three fold-consensus thresholds (40%, 60%, 80%). The 60% consensus consistently produced an intermediate number of features for most methods, balancing representativeness and stability. These results support the 60% threshold as a stable and generalizable cutoff for defining reproducible feature sets; Table S4: Overview of common survival models and their recommended clinical applications. Tree-based models such as RSF and GBS are ideal for ranking patients by risk and detecting complex interactions, while Cox and Weibull models are preferred when the goal is to estimate survival probabilities and interpret how specific factors influence outcomes.
