# Supplementary material for: Feature Selection and Model Optimization for Survival Prediction in Patients with Angina Pectoris
Source: J Clin Med. 2025 Nov 16;14(22):8111. doi: 10.3390/jcm14228111 (PMC12653679; doi:10.3390/jcm14228111)
Supplement: Supplementary file 1 [file jcm-14-08111-s001.zip › jcm-3954953-supplementary.pdf]

Feature Selection and Model Optimization for Survival Prediction in Patients with Angina Pectoris

Róbert Bata[1], Amr Sayed Ghanem[1], Attila Csaba Nagy[1,*]

[1]Department of Epidemiology, Faculty of Health Sciences, University of Debrecen, Debrecen, Hungary

[*]corresponding author: nagy.attila@etk.unideb.hu

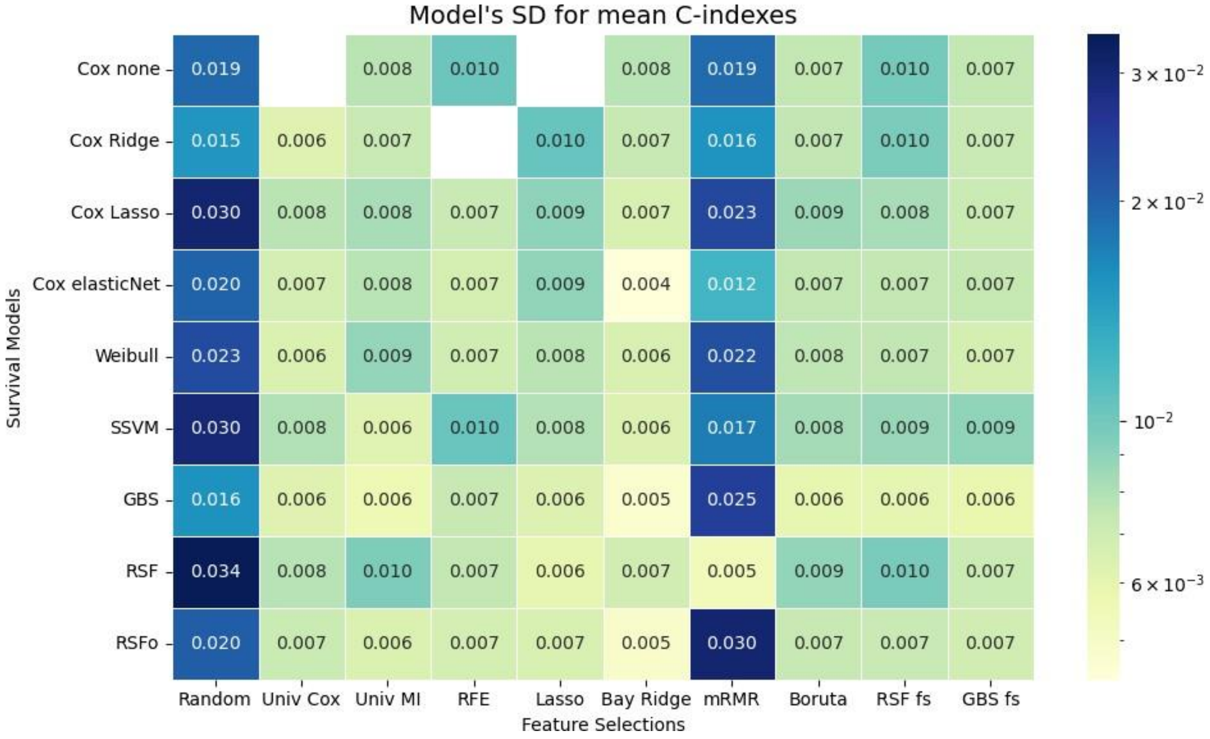

Supplementary Figure S1. *The standard deviation for the mean C-indexes.*

Feat. Sel. Method	C-index (40)	IBS (40)	C-index (60)	IBS (60)	C-index (60)	IBS (60)
**Boruta**	0.837 ±0.008	0.109 ±0.003	**0.840 ±0.009**	**0.107 ±0.003**	0.843 ±0.010	0.107 ±0.003
**LASSO**	0.747 ±0.013	0.130 ±0.001	**0.757 ±0.015**	**0.128 ±0.001**	0.762 ±0.012	0.126 ±0.001

Supplementary Table S1. *Sensitivity analysis of feature set size on model performance. Comparison of mean ± standard deviation for the concordance index (C-index) and integrated Brier score (IBS) across feature set sizes (40, 60, and 80) for the baseline Cox model using Boruta and LASSO-based feature selection. The 60-feature configuration resulted in the most favourable balance between discrimination and calibration performance, supporting its selection as the common threshold for subsequent analyses.*

Fold Consensus	Mean Features	Implication	Interpretation
**40%**	60.3 ±24.1	Large variance	Loosest threshold → includes many fold-specific, unstable features.
**60%**	**49.0 ±19.8**	**Moderate variance**	**Balanced threshold → filters unstable features but retains enough predictors (sweet spot).**
**80%**	40.8 ±18.4	Smaller set	Strictest threshold → excludes informative features, overly conservative.

Supplementary Table S2 *- Sensitivity analysis of fold-consensus thresholds on the number of retained features across all feature selection methods. The table summarizes the mean ± standard deviation of feature numbers retained at different fold-consensus thresholds (40%, 60%, 80%) used to define stable features across cross-validation folds. Increasing the consensus threshold reduced the number of retained features, indicating progressive exclusion of fold-specific variables. The 60% consensus level resulted in an intermediate and balanced subset size, supporting its selection as the baseline threshold for defining reproducible features across folds.*

Feat. Sel. Method	40% Consensus	60% Consensus	80% Consensus	Trend
**Bayesian Ridge**	65.7 ±0.6	**58.0 ±0.0**	55.0 ±0.0	Stable, slight reduction
**Boruta**	54.0 ±0.0	**50.0 ±0.0**	47.0 ±0.0	Stable
**GBS**	83.0 ±0.0	**59.0 ±0.0**	36.0 ±0.0	Sharp drop beyond 60%
**LASSO**	69.0 ±0.0	**60.0 ±0.0**	50.0 ±0.0	Moderate reduction
**RFE**	74.0 ±0.0	**55.0 ±0.0**	43.0 ±0.0	Large reduction
**RSF**	62.0 ±0.0	**60.0 ±0.0**	58.0 ±0.0	Stable
**Univariate Cox**	53.7 ±42.7	**46.7 ±39.3**	41.7 ±34.1	Variable
**Univariate MI**	45.7 ±35.5	**35.7 ±25.4**	26.7 ±21.2	Decline
**mRMR**	35.7 ±43.8	**16.3 ±18.0**	9.7 ±6.7	Steep decline

Supplementary Table S3. *The table reports the mean ± standard deviation of the number of retained features for each feature selection method across three fold-consensus thresholds (40%, 60%, 80%). The 60% consensus consistently produced an intermediate number of features for most methods, balancing representativeness and stability. These results support the 60% threshold as a stable and generalizable cutoff for defining reproducible feature sets.*

Model	What It Does Best	When to Use It	Clinical Advantage
**Cox Proportional Hazards**	Estimates how each factor affects risk over time	When the goal is to understand and explain how clinical variables (e.g., lab values, comorbidities) influence patient outcomes	Easy to interpret and report as hazard ratios; widely used in clinical literature
**Weibull Model**	Predicts time until an event (e.g., survival time) assuming a known trend	When you need smooth survival curves and time-to-event estimates for prognostic modelling	Provides clear survival probability estimates for individual patients
**Survival SVM**	Ranks patients by overall risk based on many predictors	When prioritizing patients by relative risk (e.g., identifying high-risk subgroups)	Effective in handling complex relationships when interpretability is less critical
**Gradient Boosted Survival (GBS)**	Learns complex patterns and variable interactions	When predictive accuracy and patient risk ranking are priorities	Captures subtle non-linear effects; suitable for decision-support tools
**Random Survival Forest (RSF)**	Handles many variables and captures interactions automatically	When working with high-dimensional data or uncertain variable relationships	Robust and accurate; useful for identifying risk patterns and high-risk patients

Supplementary Table S4. *Overview of common survival models and their recommended clinical applications. Tree-based models such as RSF and GBS are ideal for ranking patients by risk and detecting complex interactions, while Cox and Weibull models are preferred when the goal is to estimate survival probabilities and interpret how specific factors influence outcomes.*

Model Parameter Selection and Optimization

The selection of model parameters followed a systematic and consistent evaluation process across all survival models to ensure fair comparison and reproducibility.

For the Cox proportional hazards and Weibull models, we evaluated a range of penalization strengths ($\lambda = \{0.001, 0.01, 0.1, 1\}$) and selected the configuration that achieved the best cross-validated performance based on the concordance index and calibration metrics.

For the survival support vector machine (SVM), gradient-boosted survival (GBS), and random survival forest (RSF) models, we applied standard (default) parameter configurations. This decision was made to prevent excessive hyperparameter tuning, which could introduce additional bias or overfitting, and to maintain comparability across methods.

An additional optimized RSF model was developed using Bayesian optimization to identify the most effective combination of parameters. The best-performing configuration was as follows: n_estimators=151, max_depth=10, min_samples_split=13, min_samples_leaf=7, max_features='sqrt'.

This systematic approach ensured that all models were tuned consistently, balancing predictive performance, interpretability, and generalizability without overfitting to the study data.
